# Dysregulation of Regulatory ncRNAs and Diseases

**DOI:** 10.3390/ijms25010024

**Published:** 2023-12-19

**Authors:** Mohamed Raafat El-Gewely

**Affiliations:** Department of Medical Biology, UiT Arctic University of Norway, 9037 Tromsø, Norway; raafat.el-gewely@uit.no

Cancer was initially attributed to genetic mutations and gene alterations, which resemble genetic diseases caused by various modifications of a specific gene in the genome sequence. However, other factors can affect the phenotype without any changes to the gene sequence in cancer and numerous other diseases. The factors that can affect gene expression and, consequently, the phenotype without any alterations in the genome sequence are called epigenetics. The environmental impact on gene expression, known as gene–environment interaction, was noted very early in the history of genetics as a scientific discipline. Thus, the profound environmental impact on human diseases has been well studied. Gene expressions can be influenced by the presence of environmental inducers or repressors [1]. Although the cells of any organism are genetically identical, their functional differences in different organs are attributed to epigenetics. These epigenetic effects are caused by the activities of DNA methylases and chromatin modification enzymes, as reviewed by Jaenisch and Bird 2003 [2]. Changes in epigenetic factors may arise from the altered expressions of DNA methylation or histone modification enzymes, which can often lead to diseases, including cancer [3].

Another influential aspect of epigenetics is the action of noncoding RNAs. Unlike coding RNAs, noncoding RNAs are the most predominantly expressed RNAs in cells, accounting for about 98% of the total expressed RNA, whereas coding RNAs constitute only 2% [4]. This significant discrepancy reflects the extent to which noncoding RNAs regulate gene expressions and perform housekeeping functions in the cell. Subsequently, the dysregulation of noncoding RNA gene expression significantly affects cellular health and can lead to cancer and other diseases.

Noncoding RNAs are classified into housekeeping and regulatory ncRNAs [5]. Housekeeping ncRNAs include ribosomal (rRNA), transfer (tRNA), small nuclear (snRNA), and small nucleolar RNAs (snoRNAs). Regulatory ncRNAs are divided into circular and linear RNAs, and linear RNAs are further divided into small ncRNAs (sncRNAs) and long ncRNAs (lncRNAs). The sncRNA group includes microRNAs (miRNAs), small interfering RNAs (siRNAs), and piwi-associated RNAs (piRNAs) [5].

Also, the chemical modifications of RNA molecules, known as the epitranscriptome, by RNA-modifying proteins (RMPs) was reported to regulate various RNAs functions. The RNA modification machinery was found to be frequently altered in human cancers, highlighting the immense potential of RMPs as pharmacological targets as well as diagnostic markers [6].

In this Special Issue, we focus on examining how dysregulation of the expression of regulatory noncoding RNAs (ncRNAs) leads to different cancer types and other diseases. Potential diagnostic and therapeutic targets are frequently addressed.
